# Isolation of plasma-membrane components from cultured human pancreatic cancer cells by immuno-affinity chromatography of anti-beta 2M sepharose 6MB.

**DOI:** 10.1038/bjc.1979.250

**Published:** 1979-11

**Authors:** S. Påhlman, I. Ljungstedt-Poahlman, A. Sanderson, P. J. Ward, A. Grant, J. Hermon-Taylor

## Abstract

**Images:**


*Br. J. Cancer* (1979) **40**, 701

### ISOLATION OF PLASMA-MEMBRANE COMPONENTS FROM CULTURED HUMAN PANCREATIC CANCER CELLS BY IMMUNO-AFFINITY CHROMATOGRAPHY OF ANTI-$\beta_2$M SEPHAROSE 6MB

S. PÅHLMAN†, I. LJUNGSTEDT-PÅHLMAN†, A. SANDERSON*, P. J. WARD*, A. GRANT AND J. HERMON-TAYLOR

*From the Department of Surgery, St George's Hospital Medical School and *Blond Laboratories, Queen Victoria Hospital, East Grinstead, Sussex*

Received 28 June 1979 Accepted 23 July 1979

**Summary.—Human pancreatic exocrine adenocarcinoma cells established in tissue culture expressed both HLA and $\beta_2$-microglobulin ($\beta_2$M). Plasma-membrane components of this pancreatic cancer cell line were purified from plasma membrane fractions enriched by sucrose density-gradient centrifugation, using immunoaffinity chromatography on immobilized anti-human $\beta_2$M antibody. Both rabbit and mouse monoclonal anti-$\beta_2$M IgG were used, with a 20–25-fold overall purification of 5′-nucleotidase. The method was applicable to $5 \times 10^7$ cells and permitted the solubilization of membranes retained on the column, with the selective desorption of components not associated with $\beta_2$M before the subsequent elution at pH 3 of $\beta_2$M-associated macromolecules. The acid eluate contained one major and two minor bands in the 40–45,000 mol.-wt range with two additional enriched components of 18,000 and 22,000 dalton. A major carbohydrate-containing component of high mol. wt was also found to be associated with the pancreatic cancer-cell plasma membrane.**

ONCOFOETAL MACROMOLECULAR PRODUCTS of common solid human tumours, such as carcinoembryonic antigen, $\alpha$-fetoprotein and pancreatic oncofoetal antigen (Banwo *et al.*, 1974; Gelder *et al.*, 1978), are unlikely to be of value in the development of specific sensitive early diagnostic methods, since they are not iso-immunogenic. More information is required about the plasma-membrane components of human tumour cells which may excite recognition by the host immune system. The isolation of such components, which must precede their biochemical and immunological characterization, is associated with two central technical problems. The first is the difficulty of extracting tumour-cell plasma-membrane fractions from often sclerotic primary tumour tissue heavily infiltrated with normal host cells. The establishment of a human pancreatic cancer cell line in this laboratory (Grant *et al.*, 1979), which preserves its morphological characteristics as xenografts in immune-deficient animals, should provide an apparently homogeneous source of pancreatic cancer cell membrane. The problem then becomes the availability of sufficient starting material and the need for separation methods of greater selectivity. The present investigation is concerned with the development of an immuno-affinity procedure for the isolation of plasma membranes and their components from cultured human pancreatic cancer cells, using immobilized antibody to human $\beta_2$-microglobulin ($\beta_2$M). The method, which permits selective adsorp-

† Present address: Institute of Biochemistry, Biomedical Centre, University of Uppsala, S-751 23 Uppsala, Sweden.

tion and desorption, is likely to be applicable to other human cell populations expressing this antigen.

### MATERIALS AND METHODS

*Radiolabelling of cultured cells*

Human pancreatic exocrine adenocarcinoma cells derived from a primary lesion were cultured and harvested as previously described (Grant *et al.*, 1979). Endogenous labelling of proteins and rough endoplasmic reticulum (RER) was carried out using $^{14}$C-amino acids or $^{3}$H-uridine respectively. About 10 $\mu$Ci/$10^7$ cells of $^{14}$C-amino acids (U-$^{14}$C protein hydrolysate 50 mCi/m atom carbon, Radiochemical Centre, Amersham) was incubated overnight with almost confluent pancreatic cancer cells in 10% FCS supplemented medium (Hams F12, Flow Laboratories, U.K.). 10 $\mu$Ci/$10^7$ cells of $^{3}$H-uridine (25 Ci/mmol, Radiochemical Centre, Amersham) was incubated for 24 h with almost confluent cells under similar culture conditions. In other studies exogenous labelling of surface proteins and carbohydrates was carried out on cells in suspension using $^{125}$I lactoperoxidase–glucose oxidase (Hynes, 1973) or galactose-oxidase–$NaB^3H_4$ (Gahmberg & Andersson, 1977) respectively. Before labelling by the latter method, the cells were incubated at 37°C with 15 u/ml neuraminidase for 1 h. Both the galactose oxidase (KABI, Sweden) and neuraminidase (Boehringer, U.K.) were reported to be free of proteases (Gahmberg & Andersson, 1977). After iodination or tritiation of the cell surface, viability was always greater than 90% as determined by trypan-blue exclusion.

*Membrane preparation*

Membranes were prepared from $\sim 5 \times 10^7$ cells (about 25 mg protein). The cells were detached from the culture flasks using 0·02% EDTA (Grant *et al.*, 1979) washed with PBS and suspended in 1 ml of 1mM $NaHCO_3$ + 0·2mM $MgCl_2$ and immediately centrifuged at 500 *g* at 4°C for 5 min. The supernatant contained cytoplasmic proteins but only minute amounts of plasma membrane, since the cells became leaky but did not disintegrate under these conditions. The supernatant was discarded and the pellet resuspended in 3 ml of 1mM $NaHCO_3$ + 0·2mM $MgCl_2$ at 4°C. The cells were then immediately homogenized by 5–7 strokes with a tightly fitting Dounce homogenizer (F.T. Scientific Inst. Ltd, Tewkesbury, Glos) and transferred to 7 ml of sucrose solution to give a final concentration of 0·25M sucrose + 1·5mM EDTA in a total volume of 10 ml. Nuclei and residual whole cells were removed by centrifugation at 500 *g* at 4°C for 5 min. The supernatant was used as a crude membrane preparation for the further fractionation of plasma membrane.

*Preparative procedure*

*Sucrose-gradient centrifugation.*—Discontinuous gradients were prepared by layering 3·5 ml of 20, 35 and 45% sucrose w/w in 1mM $NaHCO_3$ + 1·5mM EDTA into 15ml tubes. Gradients were loaded with 3 ml of the crude membrane preparation ($\sim$1 mg protein/ml) and centrifuged at 100,000 *g* for at least 5 h (MSE 6 × 15 S.W. rotor). The gradients were collected from the bottom of the tube into 1–1·5ml fractions. The sucrose density of the fractions was measured with a refractometer.

*Immunoaffinity chromatography.*—Human $\beta_2$M was isolated from the urine of patients with Wilson's disease as described by Berggård & Bearn (1968) and purified to apparent homogeneity. Rabbits were immunized with 0·1 mg $\beta_2$M emulsified with Freund's adjuvant and serum obtained at intervals after boost inoculations. Sera were pooled and established as having $\beta_2$M specificity by double diffusion in gels against pure antigen; control wells with other human serum proteins were consistently negative. Specific anti-human $\beta_2$M IgG was prepared from pooled serum by DEAE chromatography and established to be free from other serum proteins by immuno-electrophoresis.

Mouse anti-human $\beta_2$M was obtained as follows: a hybridoma cell line which secreted anti-$\beta_2$M IgG was established (Kohler & Milstein, 1976) by fusion of NS1 myeloma cells with spleen cells from mice sensitized with human peripheral-blood lymphocytes. After growth in HAT medium and cloning, a line was identified which produced monoclonal anti-$\beta_2$M IgG antibody. Large-scale production of this monoclonal IgG was achieved in (BALB/c × DBA/2) $F_1$ hybrid mice by isolation of serum when large tumour growth was noted. Purification of $\beta_2$M-specific antibody was achieved by adsorption and acid elution from a column of $\beta_2$M-

substituted Sepharose 4B (2mg $\beta_2$M/g wet gel). Eluted material was identified as IgG by immunoelectrophoresis and established as monoclonal by isoelectric focusing. Anti-$\beta_2$M titres were established in a radioimmunoassay as previously described (Sanderson, 1977). Anti-$\beta_2$M antibody was coupled to Sepharose 6MB (a gift from the research department of Pharmacia Fine Chemicals AB, Sweden) after activation in this laboratory using cyanogan bromide (Axen *et al.*, 1967). In order to favour binding of the antibody to the exterior of the gel beads, the Sepharose was highly activated (0·2 g CNBr/g wet gel; Grant *et al.*, 1978).

Initially, IgG fractions of rabbit anti-human $\beta_2$M serum were coupled to CNBr-activated gel at a concentration of 2 mg antibody/g wet gel. This antibody was replaced in later studies with purified monoclonal mouse anti human $\beta_2$M substituted at 0·05 mg/g wet gel. Sample volume and column size in the chromatographic procedures varied with the different experiments, details of which are given in the legends to Fig. 2 and 3. The crude membrane sample was diluted 1:1 with the operational buffer (10mM Tris-HCl, pH 8·2, +0·1M NaCl+1·5mM EDTA) and added to the anti-$\beta_2$M Sepharose 6MB in the column. The slurry was gently mixed with a glass rod at intervals over a 1h period, after which the gel was allowed to settle for another hour. Unbound material in the supernatant was removed and the column washed overnight with operational buffer (3 ml/h). Protein-bound radioactivity in the flow-through material was counted in washed TCA precipitates. Membranes retained by the gel were solubilized with detergents, Nonidet-P40 (NP-40, BDH, U.K.), Mulgofen or Deoxycholate. 2·5% NP-40 in the operational buffer was mixed with the gel bed to a final concentration of 0·5% NP-40 and the slurry again stirred at intervals for 1 h, before elution with further operational buffer containing 0·5% NP-40. Adsorbed $\beta_2$M and components linked to $\beta_2$M were then eluted with 50mM glycine-HCl, pH 3·0, +0·5% NP-40. Control experiments with non-substituted Sepharose 6MB were also performed. Fractions were concentrated on a Minicon B15 concentrator (Amicon Corp., U.S.A.). Binding of whole cells to the anti-$\beta_2$M column was also investigated by an adsorption procedure similar to that described for the membrane fractions.

*Analytical methods*

Polyacrylamide-gel electrophoresis in sodium dodecyl sulphate (SDS-PAGE) was performed in a discontinuous buffer system essentially as described by Neville (1971). Slab gels with a 4% stacking gel and a 11% separation gel were used. Samples containing 10–100 $\mu$g protein in 80mM boric acid, 80mM Tris buffer (pH 8·64) were reduced with 0·5% $\beta$-mercaptoethanol in the presence of 0·02% EDTA, 0·3% SDS and 5% sucrose, and boiled for 1 min. Electrophoresis was carried out for 4 h at a maximum of 4 W. The gels were then fixed in 20% sulphosalicylic acid, stained with 0·05% Coomassie BB R-250 in methanol–acetic-acid–water (50:7:43) and destained in methanol–acetic-acid–water (25:7:68). $^{125}$I-labelled proteins in dried gels were visualized autoradiographically; $^{3}$H- and $^{14}$C-labelled components were fluorographed according to the method of Laskey & Mills (1975). The Pre-sensitized X-ray films, X-Omate H (Kodak), were exposed for 1–4 weeks. Protein was estimated by the Lowry method in the presence of SDS. Bovine serum albumin was used as standard. 5′ nucleotidase activity of the membrane preparations was determined according to Avruch & Wallach (1971) using [$^{14}$C]AMP (Amersham) as tracer radioactivity. For this assay and for all other radioactive samples in water, a scintillation cocktail consisting of 1 l toluene, 0·5 l Triton X-100, 5 g PPO and 50 mg POPOP was used.

*Immunofluorescent staining of cells for HLA and $\beta_2$M*

Suspensions of viable cultured human pancreatic cancer cells ($10^6$ cells/100 $\mu$l PBS) were incubated with monoclonal mouse anti-human HLA W6/32 (a gift from Dr A Williams; Barnstable *et al.*, 1978) and anti-$\beta_2$M antibodies at room temperature for 1 h. The cells were washed twice with PBS, re-suspended in 100 $\mu$l and labelled with 10–20 $\mu$l FITC- or rhodamine-conjugated anti-mouse or anti-rabbit IgG (Nordic Immunological Laboratories, Maidenhead) for 30 min at room temperature. In control experiments monoclonal mouse anti-rat AgB or normal rabbit serum respectively were used. Cells were mounted in PBS glycerol (1:1 v/v) and examined with a Zeiss ultraphot IIIb with epi-fluorescent illumination.

### RESULTS

### *Membrane preparation and sucrose-gradient centrifugation*

The cultured human pancreatic cancer cells were noticeably more resistant to disruption by hypotonic medium and Dounce homogenization than human lymphocytes or fibroblasts (Graham, 1975). 0·2mM $Mg^{2+}$ was required during homogenization to prevent nuclear rupture, but when the cell homogenate was transferred to isotonic sucrose, EDTA became necessary to prevent membrane aggregation associated with the presence of $Mg^{2+}$ (Avruch & Wallach, 1971). Under these circumstances the yield was in the range 65–75% of available plasma membrane as assessed by 5′nucleotidase activity; omission of $Mg^{+2}$ and EDTA reduced the membrane yield to only 20–25%.

The results of sucrose-gradient centrifugation are shown in Fig. 1. With $^3$H-labelled surface glycoproteins, a major peak of radioactivity which banded on top of the 35% sucrose coincided with a protein and 5′nucleotidase peak (Fig. 1A). The radioactivity on top of the sucrose gradient (Fractions 1–4, Fig. 1A) is unbound $^3$H which penetrated the intact cells during the labelling procedure. This could be removed by dialysis after homogenization, since subsequent SDS-PAGE of these fractions and fluorography showed no radioactivity associated with macromolecules.

Fig. 1B shows the results obtained with $^{14}$C amino acid-labelled cells. The peak of radioactivity in Fraction 1–4 corresponds to the similar protein peak in Fig. 1A, and represents cytoplasmic proteins and some free labelled amino acids. The 5′nucleotidase activity does not precisely coincide with the distribution of radioactivity, suggesting the presence of other components in the plasma-membrane fractions. The results shown in Fig. 1C with labelled RNA suggest that the major 5′nucleotidase peak is contaminated with RER. In all 3 experiments a second plasma-

FIG. 1.—Discontinuous sucrose-gradient centrifugation. 3 ml of crude membranes (~1 mg protein/ml) was layered on to the following gradient: 3·5 ml 20%, 3·5 ml 35% and 3·0 ml 45% (w/w) sucrose (see Methods). The crude-membrane samples were prepared from cells labelled with either (A) galactose-oxidase–$NaB^3H_4$, (B) $^{14}$C-amino acids, or (C) $^3$H-uridine. The fractions (~1·5 ml) were analysed for radioactivity (▲—▲), protein content (●—●), sucrose density (×—×) and 5′nucleotidase activity (□—□). The protein and sucrose density patterns shown in A are similar in B and C.

membrane peak was detected, banding on top of the 45% sucrose zone. The amount of material varied from one experiment to another, but visual observation suggested that this fraction consisted of aggregated membranes. The overall enrichment of the plasma-membrane fractions represented only a 4–5-fold purification and, with the limited amount of starting material available, it was clear that a preparative procedure of greater selectivity was necessary.

*Anti-$\beta_2$M-Sepharose 6MB column chromatography*

At least 95% of the cultured pancreatic cancer cells stained for both HLA and $\beta_2$M by indirect immunofluorescence. Both mouse monoclonal and rabbit anti-human $\beta_2$M were available in sufficient quantity to prepare respective immunoadsorbents. In the experiments using rabbit IgG, gels were substituted with ~2 mg antibody per g wet agarose. Higher substitution would not, according to Weston & Scorer (1978), increase the capacity of the gel, and might increase nonspecific adsorption (Titanji & Påhlman, 1978). Since the membranes were unlikely to penetrate the 6% agarose beads, coupling conditions were chosen which favoured substitution on the exterior of the beads (Grant *et al.*, 1978). The monoclonal antibody preparation had an approximately 40 times higher titre than the rabbit anti-$\beta_2$M antibody. Gels with monoclonal antibodies were substituted with 50 $\mu$g antibody per g gel.

The elution profile of the crude membrane preparation with labelled surface glycoproteins on monoclonal anti-$\beta_2$M Sepharose 6MB column is shown in Fig. 2. Elution of bound material with 0·5% NP-40 brought off a peak of radioactivity which contained 5′nucleotidase activity. Subsequent elution at pH 3 desorbed a trace of protein, which after concentration was visualized by SDS-PAGE (Fig. 4). NP-40 was found to be the most suitable

FIG. 2.—Purification of plasma membrane components on anti-$\beta_2$M Sepharose 6MB. 5 ml of crude membranes (~1 mg protein/ml) with labelled surface glycoproteins (galactose oxidase-$NaB^3H_4$) were applied to a 10ml gel bed (6 × 1·5 cm) substituted with mouse monoclonal antibody. Retained material was eluted with 0·5% NP-40 in the operational buffer followed by 50mM glycine-HCl (pH 3) + 0·5% NP-40. Flow rate was 3 ml/h; 3ml fractions were analysed for radioactivity (▲—▲) and 5′nucleotidase activity (□—□).

of the 3 detergents tested, and did not affect 5'nucleotidase activity. Mulgofen was as efficient as NP-40 in desorbing the plasma membrane proteins, but inactivated 5'nucleotidase over a 24h period. Deoxycholate was less efficient than NP-40 and activated 5'nucleotidase about 1·5-fold.

A 10ml bed of anti-$\beta_2$M-substituted gel retained 25–40 $\mu$g of protein. In the experiment shown in Fig. 2, about 60% of the applied 5'nucleotidase activity did not bind to the gel and when this flow-through material was rechromatographed on a fresh anti-$\beta_2$M column only a small fraction was retained. The same phenomenon was seen with intact tumour cells, since about 40% of cells applied consistently failed to bind to the anti-$\beta_2$M column, despite positive staining of the unbound population for surface $\beta_2$M by immunofluorescence. The proportion of both membranes and cells which failed to bind increased if adsorption was carried out as a batch procedure. About 8% of $^{125}$I-labelled membranes were retained when applied to a column of unsubstituted sepharose 6MB, and 4% when mixed with unsubstituted gel in a batch procedure. This suggests that only a small proportion of the adsorbed material in the column experiments was due to the physical entrapment of aggregated or large membrane fragments in the packed gel.

The selectivity of the immuno-affinity chromatography with respect to RER is shown in Fig. 3. In two separate experiments, under identical conditions, crude membrane preparations with either $^{125}$I-labelled plasma membranes or $^{3}$H-uridine-labelled RER were chromatographed on anti-$\beta_2$M Sepharose 6MB. Desorption with the nonpolar detergent Mulgofen eluted a peak of $^{125}$I-labelled plasma membranes, but only a trace of $^{3}$H-labelled RER. The proportion of RER (9%) that eluted with detergent was similar to the proportion retained nonspecifically to unsubstituted gel, suggesting physical entrapment as previously described.

FIG. 3.—Selectivity of the immuno-affinity technique. 4·5ml aliquots of a crude-membrane preparation (~0·4 mg protein/ml) were applied to 5ml gel beds (6·7 × 1·0 cm) substituted with rabbit anti-$\beta_2$M antibody. Flow rate was 3 ml/h and 2ml fractions were analysed for radioactivity. (▲—▲) cells labelled with $^{3}$H-uridine. (○—○) Cell surface labelled with $^{125}$I.

Fig. 4A shows the protein stain of an SDS-PAGE of crude membranes and fractionated plasma membrane components. Fig. 4B shows the fluorograph of the same gel, the glycoproteins of which were labelled by the galactose-oxidase–sodium-borotritide method. Fig. 4C is a fluorograph of the $^{14}$C-amino-acid-labelled plasma-membrane-enriched fraction. A progressive selection of components from the crude membrane preparation is seen as fractionation proceeds. The acid-eluted material from the anti-$\beta_2$M column contains one major and two minor components in the 40–45,000 mol. wt range; two other enriched components of ~mol. wt 22,000 and 18,000 are seen in this fraction (Fig. 4A, lane 4). The most striking feature of the corresponding fluorograph is the major radioactive component trapped on top of the 4% stacking gel and present in all fractions including

FIG. 4.—SDS-PAGE followed by fluorography of fractionated plasma-membrane components. (A) Slab gel stained for protein—Lane 1: Crude membrane preparation from cells labelled with galactose-oxidase–$NaB^3H_4$. Lane 2: The plasma-membrane-enriched fraction from the sucrose gradient in Fig. 1A. Lane 3: NP-40-eluted material from α-$\beta_2$M column shown in Fig. 2. Lane 4: pH3-eluted material from α-$\beta_2$M column shown in Fig. 2. Protein standards: human serum albumin (204,000, 136,000 and 68,000), human IgG (50,000 and 25,000), ovalbumin (43,000) and cytochrome c (13,800). (B) Corresponding fluorograph. (C) Fluorography of the $^{14}$C amino acid-labelled plasma-membrane-enriched fraction (Fraction 6) from the sucrose gradient shown in Fig. 1B (comparable with Lane 2 of (A)).

the NP-40 eluate from the affinity column, but absent from the acid-eluted fraction. This high-mol.-wt surface component which does not appear in the protein stain and contains little incorporated $^{14}$C-amino acid (Fig. 4A, 1–3 and Fig. 4C, respectively) must be highly glycosylated. Radio-labelling of other membrane glycoproteins with galactose-oxidase–$NaB^3H_4$ was poor (Fig. 4B).

### DISCUSSION

The human pancreatic exocrine cancer cell line previously described (Grant *et al.*, 1979) clearly expresses both the HLA antigens and $\beta_2$M in conditions of *in vitro* culture and, despite the abnormalities in chromosome content, has functional Chromosomes 6 and 15. The expression of these components is preserved during serial passage. In Daudi, by contrast, a human lymphoblastoid cell line Chromosome 15 is missing and 6 is cryptic (Goodfellow *et al.*, 1975). However, Daudi grows well in suspension culture, whereas the pancreatic cancer cells, like cell lines derived from other solid tumours, require a matrix for growth. This limits the number of cells that can be made available for study, and means that preparative procedures for the isolation of their plasma membranes must combine maximum resolution with high yield, conditions which are difficult to attain with centrifugation techniques alone.

The expression of $\beta_2$M at the cell surface, and the availability of sufficient specific antibody as a ligand, permitted the development of an affinity chromatography system for the selective absorption plasma membranes. This had the additional advantage of offering selective desorption from the column of membrane-bound macromolecules not associated with $\beta_2$M before the elution of $\beta_2$M-containing histocompatibility antigens and other components which might be associated with the malignant state (Kvist *et al.*, 1978; Tada *et al.*, 1978; Beutler *et al.*, 1978). Similar methods have been applied to the isolation from solid tissue of papain-solubilized $\beta_2$M-associated components in human malignant melanoma, breast carcinoma, colonic carcinoma and hepatoma (Thomson *et al.*, 1978; Rauch *et al.*, 1978).

Despite the observation that at least 95% of the cultured tumour cells were positive by immunofluorescent staining for $\beta_2$M, only 50% of the plasma membranes were apparently able to bind to the gel. It seems unlikely that this could be due to the formation of inside-out plasma-membrane vesicles, itself an unusual event, since a similar fraction of whole cells remained unbound. It is more likely to be due to heterogeneity at the cell surface in the density of $\beta_2$M expression or differences in its accessibility to free as opposed to CNBr-immobilized Ig. Similar unbound cell populations have been described in the lectin-affinity chromatography of rabbit thymocyte membranes (Brunner *et al.*, 1977) and in the affinity separation of lymphocytes on immobilized insulin (Ljungsted-Påhlman *et al.*, 1977).

The specificity of the immunoadsorbent appeared to be high. The trace of RER retained could either be due to physical entrapment as a result of aggregation, or to ionic and hydrophobic interactions with immobilized Ig (Titanji & Påhlman, 1978). The small amount of material available and the interference of detergent in the assays made it difficult to quantify the purification achieved, but it was of the order of 10-fold for the affinity step alone with an overall purification of 20–25-fold.

It has been suggested that tumour-specific macromolecules may be associated with $\beta_2$M and HLA antigens (Kvist *et al.*, 1978; Tada *et al.*, 1978; Beutler *et al.*, 1978; Thomson *et al.*, 1978; Rauch *et al.*, 1978). The components eluted from the immuno-absorbant at pH 3 resemble similar material isolated by Thomson *et al.* (1978) from the solid tissue of other human tumours. The biological significance of the components derived from the pancreatic cancer cell line in the present study awaits the application of suitably specific cytological assays.

During the initial steps in the preparation of membranes, the cultured human pancreatic cancer cells were found to be much more resistant to disruption in a hypotonic environment than peripheral-blood lymphocytes or cultured human fibroblasts (Graham, 1975). Under hypotonic conditions the cells became leaky of cytoplasmic components, presumably due to defects in the plasma membrane, but the peripheral envelope itself did not disintegrate. We also found that the pancreatic cancer cell surface labelled poorly with galactose-oxidase–$NaB^3H_4$ and $^{125}$I-lactoperoxidase, the only exception being the large glycosylated component seen on top of the 4% stacking gel in the SDS-PAGE. It is probable that this component contributes to a substantial glycocalyx which might be responsible for some structural stabilization of the plasma membrane. It might also serve to protect the cell surface from enzymic modification and immunological attack.

We thank Dr Alan Williams for the W6/32 monoclonal antibody, and Dr J Graham (Department of Biochemistry) and Dr C. Wylie (Department of Structural Biology) at St George's Hospital Medical School for their help. Dr J. Walshe of Addenbrooke's Hospital, Cambridge, kindly provided the $\beta_2$-microglobulin-containing urine. This investigation was supported by a grant from the Cancer Research Campaign. Drs S. and I. Påhlman were funded by travelling fellowships from "OE och Edla Johanssons vetenskapliga stiftelse", "Byråchefen Stenholms stipendiestiftelse", "Gålo stiftelsen", "Apotekarsocietetens forskarstipendium" and "IF stiftelse för farmaceutisk forskning".

### REFERENCES

AVRUCH, J. & WALLACH, D. F. H. (1971) Preparation and properties of plasma membrane and endoplasmic reticulum fragments from isolated rat fat cells. *Biochim. Biophys. Acta*, **233**, 334.

AXEN, R., PORATH, J. & ERNBACK, S. (1967) Chemical coupling of peptides and proteins to polysaccharides by means of cyanogen halides. *Nature*, **214**, 1302.

BANWO, O., VERSEY, J. & HOBBS, J. R. (1974) New oncofetal antigen for human pancreas. *Lancet*, i, 643.

BARNSTABLE, C. J., BODMER, W. F., BROWN, G. & 4 others (1978) Production of monoclonal antibodies to group A erythrocytes, HLA and other tumour cell surface antigens—New tools for genetic analysis. *Cell*, **14**, 9.

BERGGÅRD, J. & BEARN, A. G. (1968) Isolation and properties of a low molecular weight $\beta_2$-globulin occurring in human biological fluids. *J. Biol. Chem.*, **243**, 4095.

BEUTLER, B., NAGAI, Y., OHNO, S., KLEIN, G. & SHAPIRO, I. M. (1978) The HLA-dependent expression of testis-organising H-Y antigen by human male cells. *Cell*, **13**, 509.

BRUNNER, G., FERBER, E. & RESCH, K. (1977) Fractionation of membrane vesicles: 1. A separation method for different populations of membrane vesicles of thymocytes by affinity chromatography on Con A-sepharose. *Analyt. Biochem.*, **80**, 420.

GAHMBERG, C. G. & ANDERSSON, L. C. (1977) Selective radioactive labelling of cell surface sialoglycoproteins by periodate-tritiated borohydride. *J. Biol. Chi.*, **252**, 5888.

GELDER, F. B., REESE, C. J., MOOSA, A. R., HALL, T. & HUNTER, R. (1978) Purification, partial characterisation and clinical evaluation of a pancreatic oncofetal antigen (POA). *Cancer Res.*, **38**, 313.

GOODFELLOW, P. N., JONES, E. A., VAN HEYNINGEN, V. & 4 others (1975) The $\beta_2$-microglobulin gene is on chromosome 15 and not in the HL-A region. *Nature*, **254**, 267.

GRAHAM, J. (1975) Cell membrane fractionation. In *New Techniques in Biophysics and Cell Biology*. Eds. Rayne & Smith. **2**, 1.

GRANT, A. G., DUKE, D. & HERMON-TAYLOR, J. (1979) Establishment and characterisation of primary human pancreatic carcinoma in continuous culture and in nude mice. *Br. J. Cancer*, **39**, 143.

GRANT, D. A. W., MAGEE, A. I. & HERMON-TAYLOR, J. (1978) Optimisation of conditions for the affinity chromatography of human enterokinase on immobilised p.aminobenzamidine: Improvement of the preparative procedure by inclusion of negative affinity chromatography with glycylglycyl-aniline. *Eur. J. Biochem.*, **88**, 183.

HYNES, R. O. (1973) Alteration of cell surface proteins by viral transformation and proteolysis. *Proc. Natl Acad. Sci. U.S.A.*, **70**, 3170.

KOHLER, G. & MILSTEIN, C. (1976) Derivation of specific antibody-producing tissue culture and tumour lines by cell fusion. *Eur. J. Immunol.*, **6**, 511.

KVIST, S., ÖSTBERG, L., PERSSON, H., PHILIPSON, L. & PETERSON, P. A. (1978) Molecular association between transplantation antigens and cell surface antigen in an adenovirus-transformed cell line. *Proc. Natl Acad. Sci.*, **75**, 5674.

LASKEY, R. A. & MILLS, A. D. (1975) Quantitative film detection of $^3$H and $^{14}$C in polyacrylamide gels in fluorography. *Eur. J. Biochem.*, **56**, 335.

LJUNGSTEDT-PÅHLMAN, I., SEIVING, B. & SJÖHOLM, I. (1977) Heterogeneous insulin and concanavalin A-binding among spleen lymphocytes established by affinity chromatography. *Exp. Cell. Res.*, **110**, 191.

NEVILLE, D. M. (1971) Molecular weight determination of protein-dodecyl sulfate complexes by gel electrophoresis in a discontinuous buffer system. *J. Biol. Chem.*, **246**, 6328.

RAUCH, J. E., SHUSTER, J., THOMSON, D. H. P. & GOLD, P. (1978) Isolation of HLA and tumour antigens by means of affinity chromatography employing anti-$\beta_2$ microglobulin antiserum. *Cancer*, **42**, 1601.

SANDERSON, A. R. (1977) HLA "help" for human $\beta_2$-microglobulin across species barriers. *Nature*, **269**, 414.

TADA, N., TANIGAKI, N. & PRESSMAN, D. (1978) Human cell membrane components bound to $\beta_2$ microglobulin in T cell-type cell lines. *J. Immunol.*, **120**, 513.

THOMSON, D. M. P., RAUCH, J. E., WEATHERHEAD, J. C., FRIEDLANDER, P., O'CONNOR, R., GROSSER, N., SHUSTERN, J. & GOLD, P. (1978) Isolation of human tumour-specific antigens associated with $\beta_2$ microglobulin. *Br. J. Cancer*, **37**, 753.

TITANJI, V. P. K. & PÅHLMAN, S. (1978) Protamine-agarose and non-charged alkyl derivatives of agarose in the purification of rat-liver phosphoprotein phosphatases. *Biochim. Biophys. Acta*, **525**, 380.

WESTON, P. D. & SCORER, R. (1978) Affinity chromatography. *Proc. Int. Symp., Vienna 1977*. (Eds. Hoffman-Ostenhof *et al.*) Oxford: Pergamon Press. p. 161.


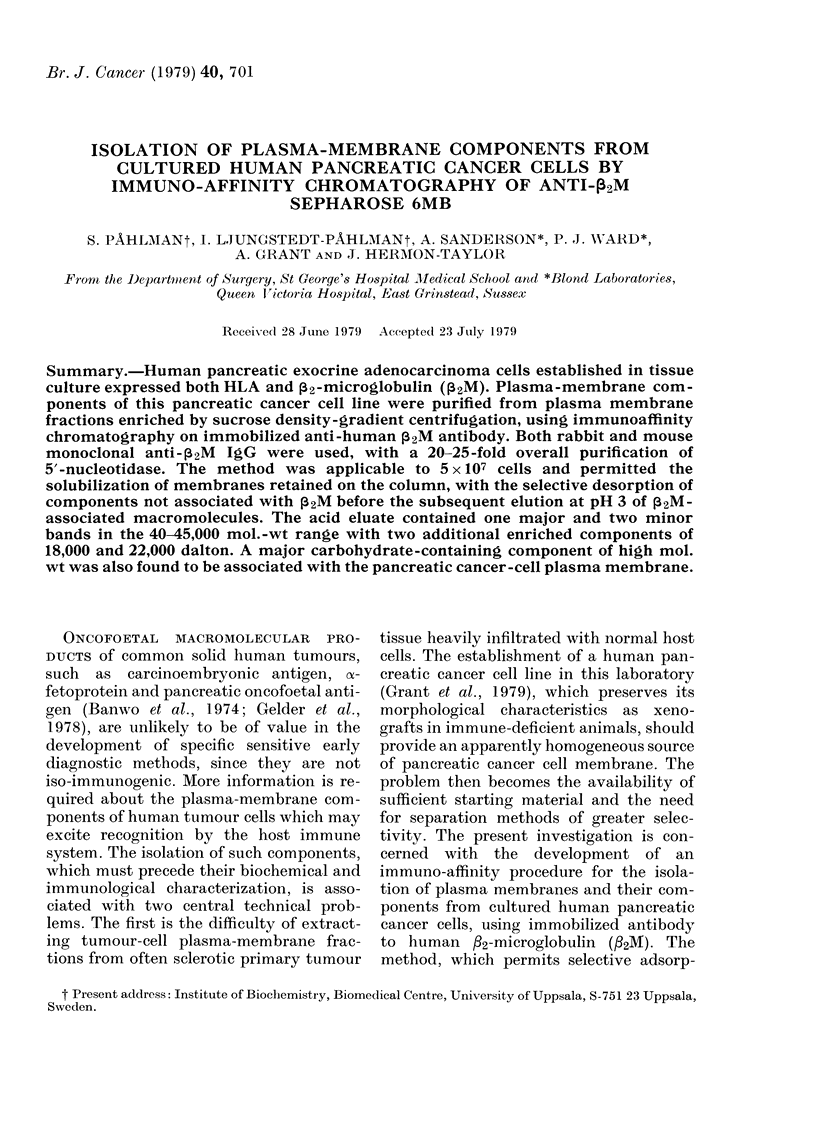

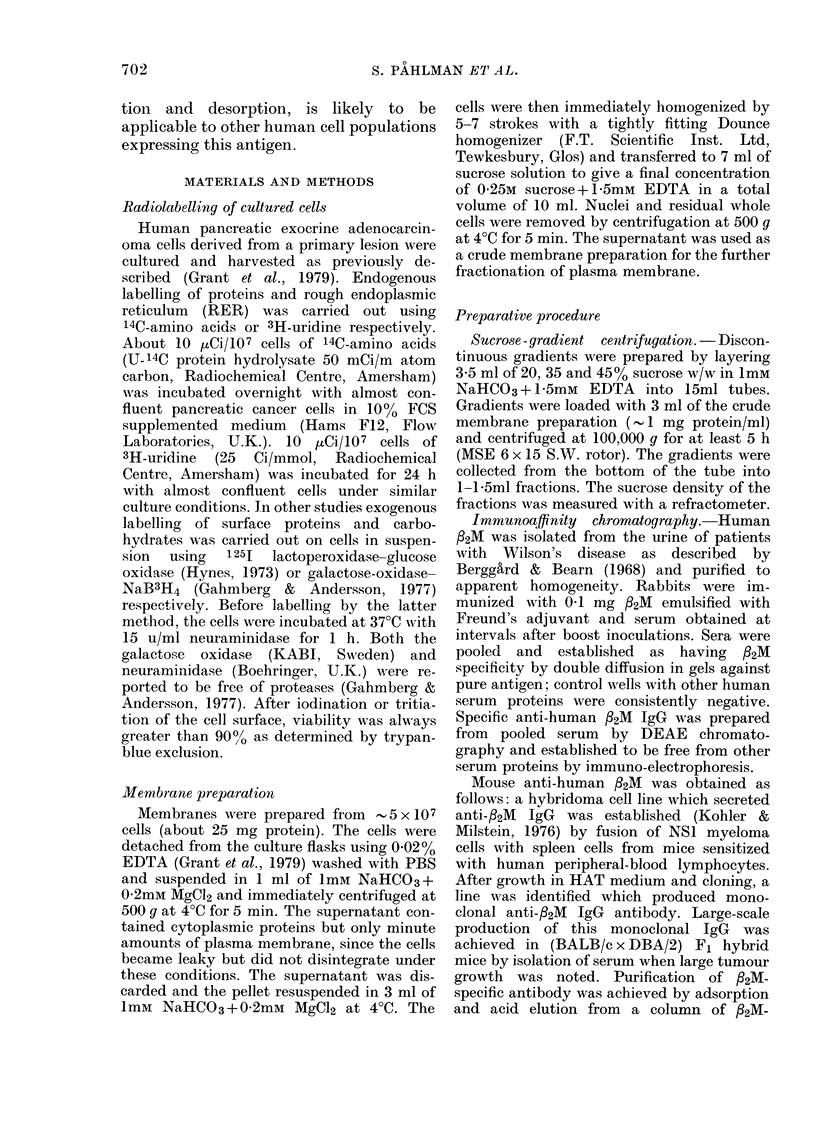

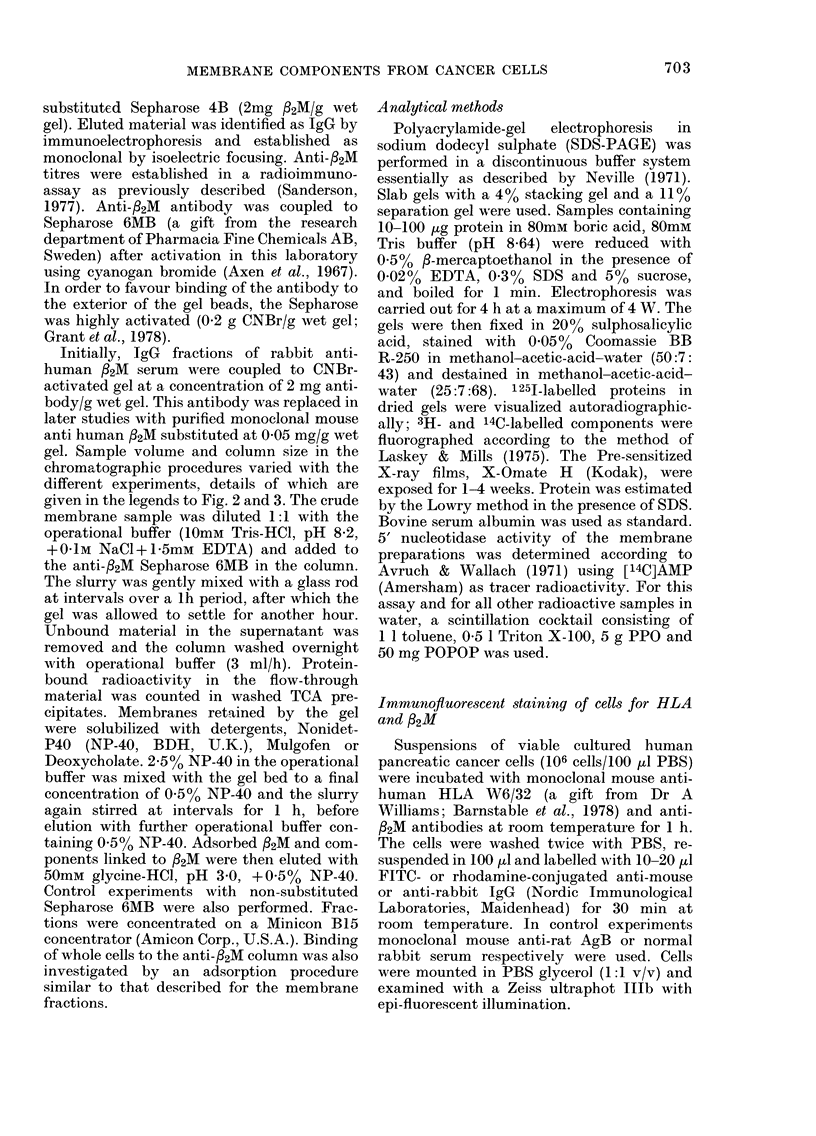

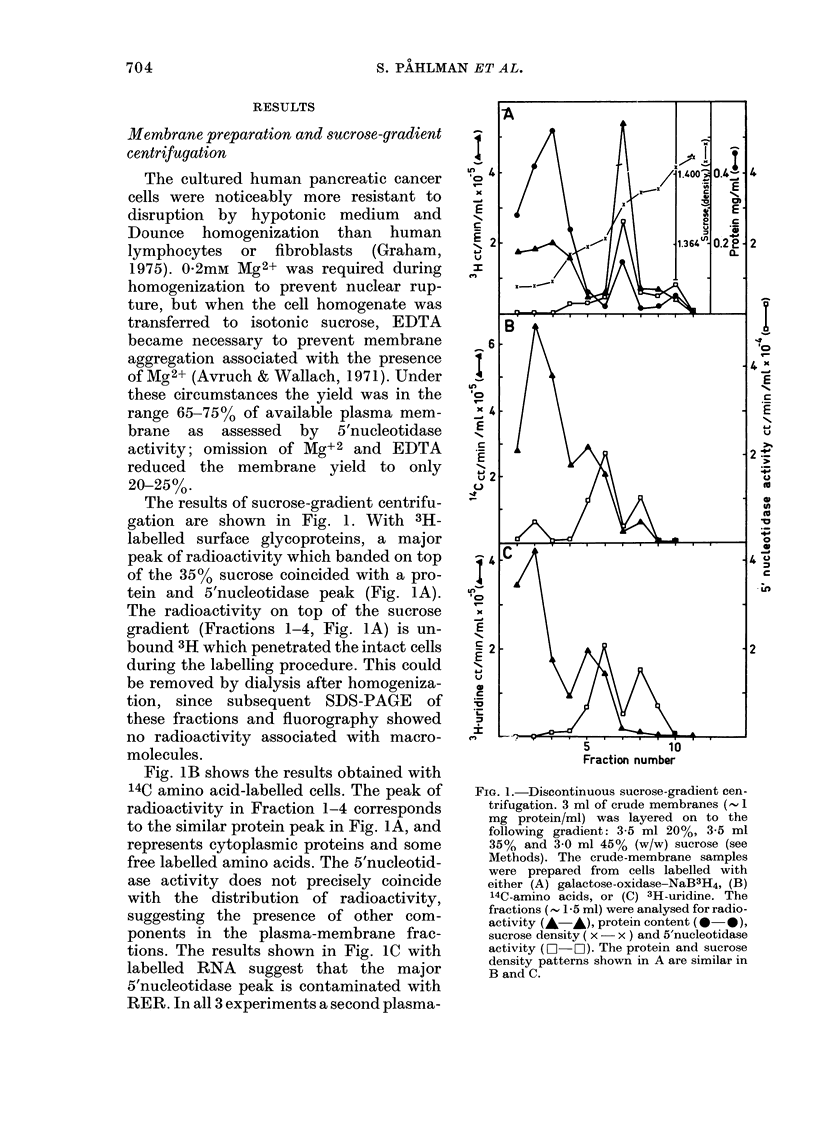

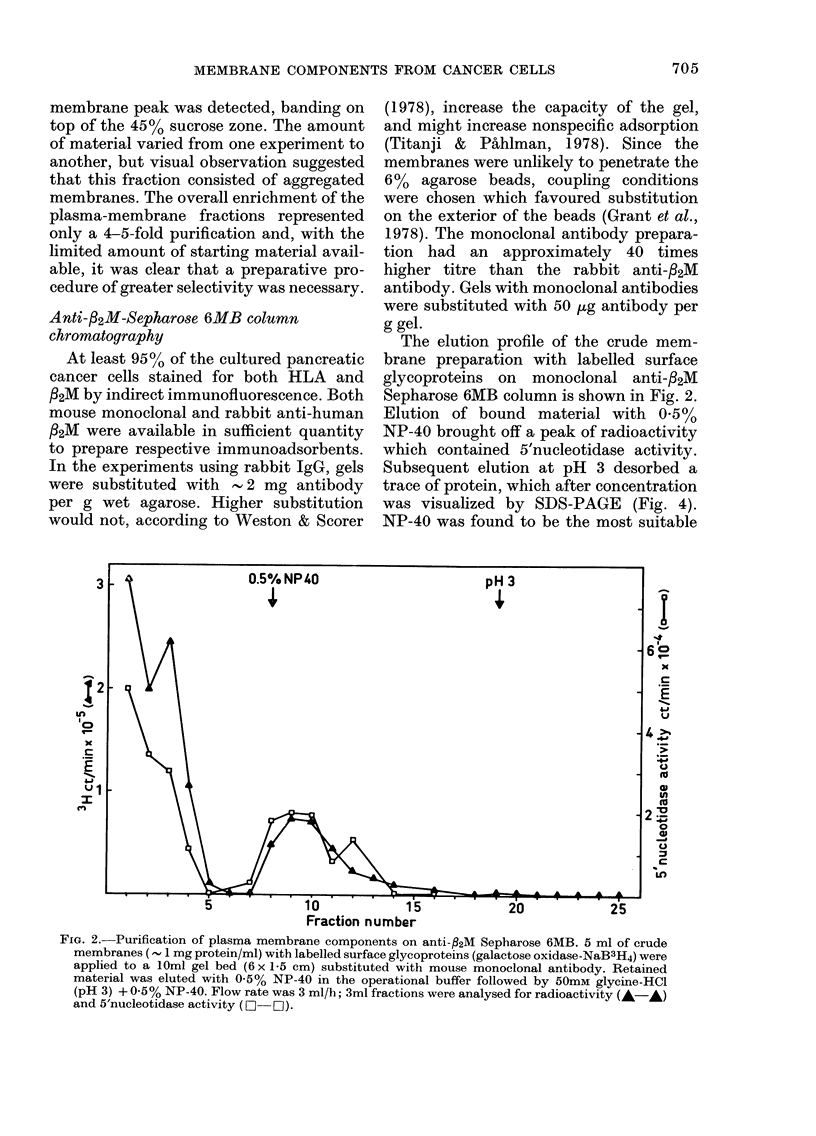

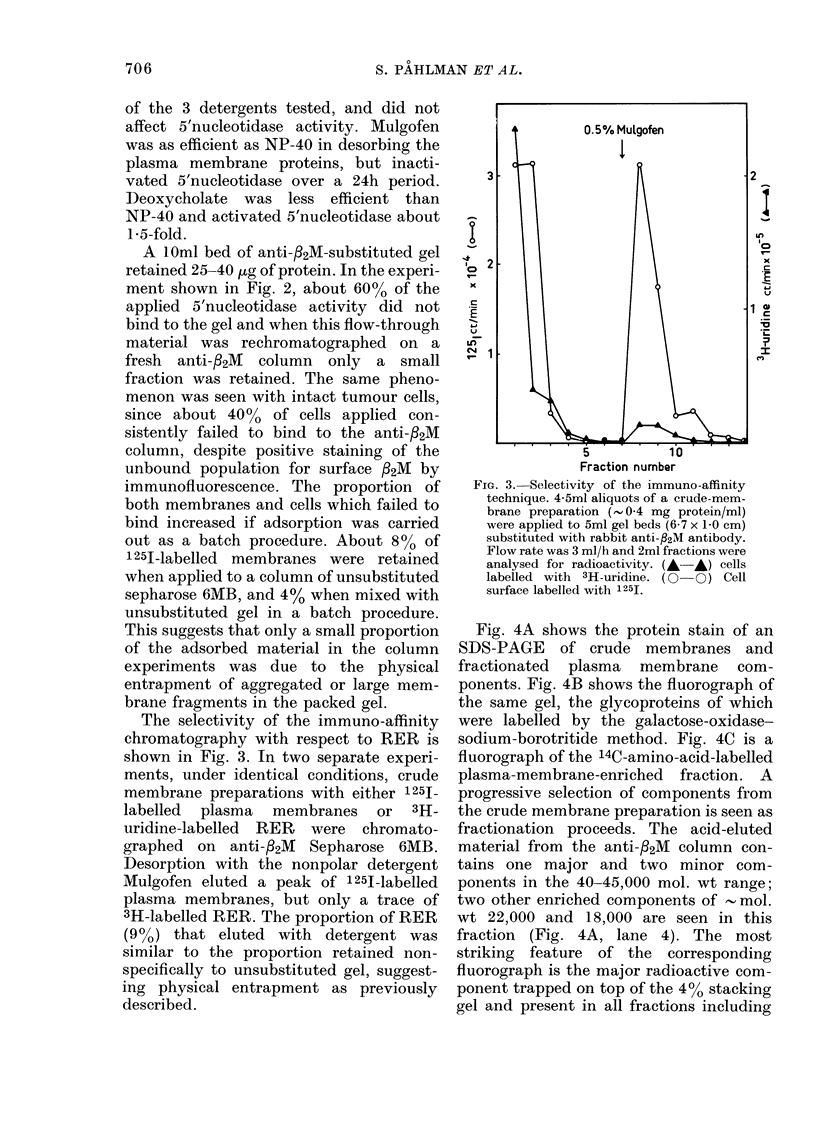

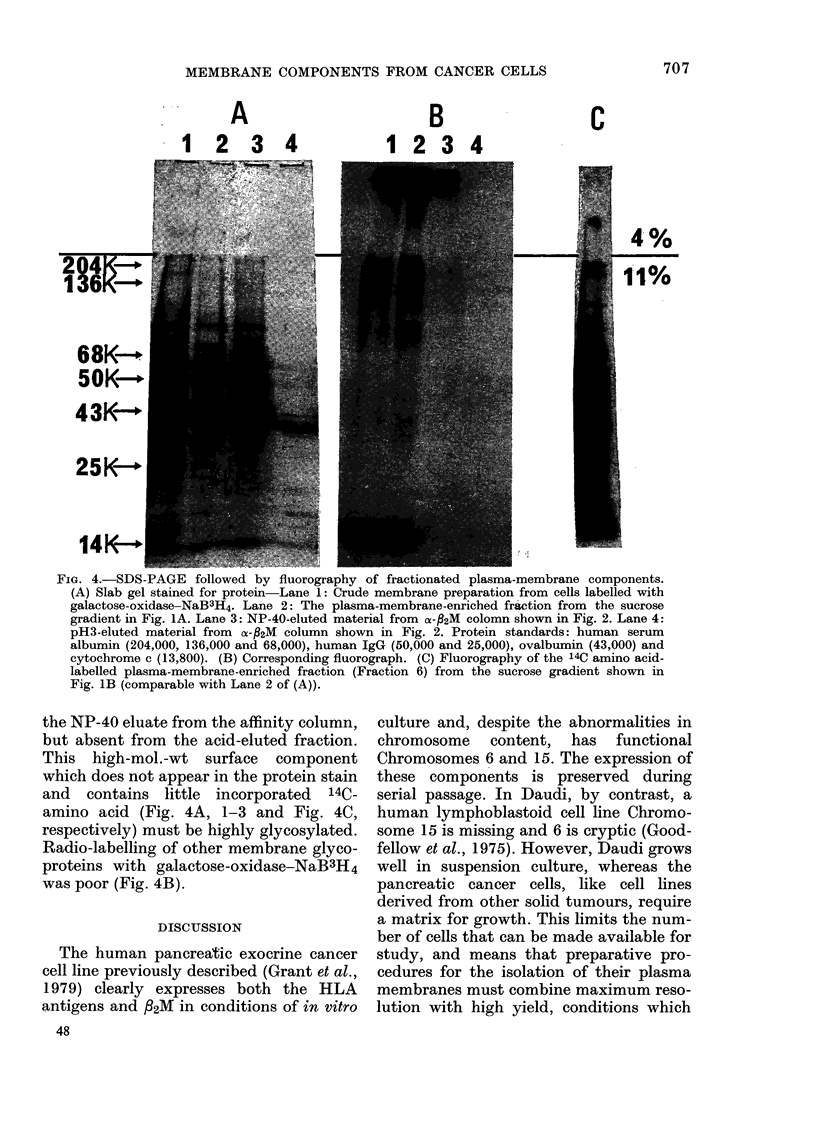

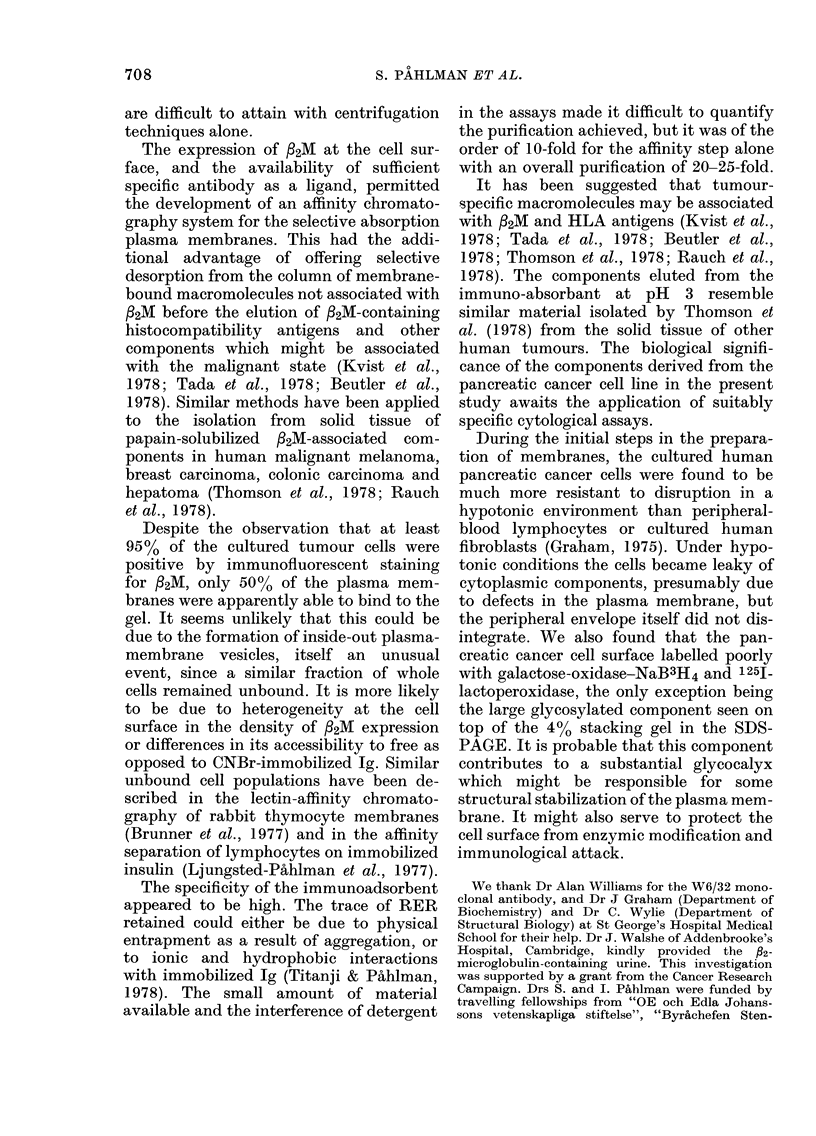

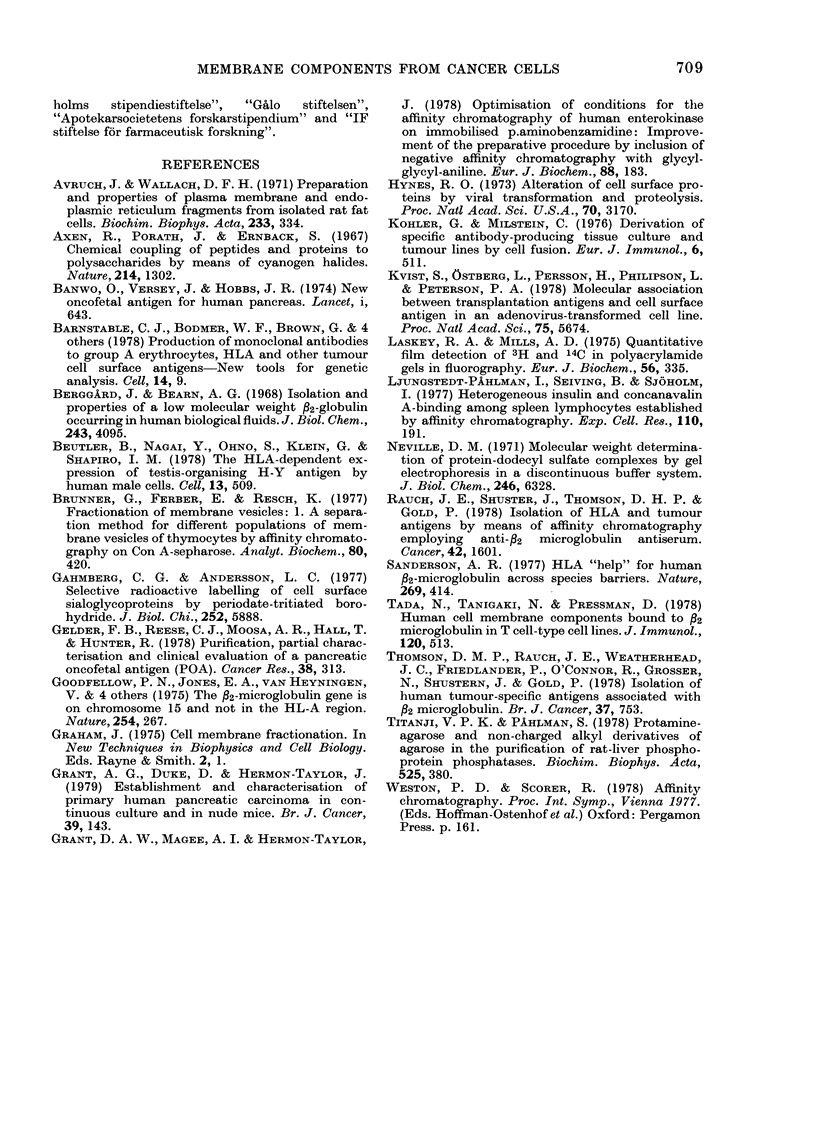


## References

[OCR_00831] Avruch J., Wallach D. F. (1971). Preparation and properties of plasma membrane and endoplasmic reticulum fragments from isolated rat fat cells.. Biochim Biophys Acta.

[OCR_00837] Axén R., Porath J., Ernback S. (1967). Chemical coupling of peptides and proteins to polysaccharides by means of cyanogen halides.. Nature.

[OCR_00843] Banwo O., Versey J., Hobbs J. R. (1974). New oncofetal antigen for human pancreas.. Lancet.

[OCR_00855] Berggård I., Bearn A. G. (1968). Isolation and properties of a low molecular weight beta-2-globulin occurring in human biological fluids.. J Biol Chem.

[OCR_00861] Beutler B., Nagai Y., Ohno S., Klein G., Shapiro I. M. (1978). The HLA-dependent expression of testis- organizing H-Y antigen by human male cells.. Cell.

[OCR_00867] Brunner G., Ferber E., Resch K. (1977). Fractionation of membrane vesicles. I. A separation method for different populations of membrane vesicles of thymocytes by affinity chromatography on Con A-Sepharose.. Anal Biochem.

[OCR_00875] Gahmberg C. G., Andersson L. C. (1977). Selective radioactive labeling of cell surface sialoglycoproteins by periodate-tritiated borohydride.. J Biol Chem.

[OCR_00881] Gelder F. B., Reese C. J., Moossa A. R., Hall T., Hunter R. (1978). Purification, partial characterization, and clinical evaluation of a pancreatic oncofetal antigen.. Cancer Res.

[OCR_00887] Goodfellow P. N., Jones E. A., Van Heyningen V., Solomon E., Bobrow M., Miggiano V., Bodmer W. F. (1975). The beta2-microglobulin gene is on chromosome 15 and not in the HL-A region.. Nature.

[OCR_00898] Grant A. G., Duke D., Hermon-Taylor J. (1979). Establishment and characterization of primary human pancreatic carcinoma in continuous cell culture and in nude mice.. Br J Cancer.

[OCR_00905] Grant D. A., Magee A. I., Hermon-Taylor J. (1978). Optimisation of conditions for the affinity chromatography of human enterokinase on immobilised p-aminobenzamidine. Improvement of the preparative procedure by inclusion of negative affinity chromatography with glycylglycyl-aniline.. Eur J Biochem.

[OCR_00914] Hynes R. O. (1973). Alteration of cell-surface proteins by viral transformation and by proteolysis.. Proc Natl Acad Sci U S A.

[OCR_00925] Kvist S., Ostberg L., Persson H., Philipson L., Peterson P. A. (1978). Molecular association between transplantation antigens and cell surface antigen in adenovirus-transformed cell line.. Proc Natl Acad Sci U S A.

[OCR_00919] Köhler G., Milstein C. (1976). Derivation of specific antibody-producing tissue culture and tumor lines by cell fusion.. Eur J Immunol.

[OCR_00932] Laskey R. A., Mills A. D. (1975). Quantitative film detection of 3H and 14C in polyacrylamide gels by fluorography.. Eur J Biochem.

[OCR_00937] Ljungstedt-Påhlman I., Seiving B., Sjöholm I. (1977). Heterogeneous insulin- and concanavalin A-binding among spleen lymphocytes established by affinity chromatography.. Exp Cell Res.

[OCR_00944] Neville D. M. (1971). Molecular weight determination of protein-dodecyl sulfate complexes by gel electrophoresis in a discontinuous buffer system.. J Biol Chem.

[OCR_00950] Rauch J. E., Shuster J., Thomson D. M., Gold P. (1978). Isolation of HLA and tumor antigens by means of affinity chromatography employing anti-beta2-microglobulin (beta2m) antiserum.. Cancer.

[OCR_00957] Sanderson A. R. (1977). HLA "help" for human B2-microglobulin across species barriers.. Nature.

[OCR_00962] Tada N., Tanigaki N., Pressman D. (1978). Human cell membrane components bound to beta2-microglobulin in T cell-type cell lines.. J Immunol.

[OCR_00968] Thomson D. M., Rauch J. E., Weatherhead J. C., Friedlander P., O'Connor R., Grosser N., Shuster J., Gold P. (1978). Isolation of human tumour-specific antigens associated with beta2 microglobulin.. Br J Cancer.

[OCR_00975] Titanji V. P., Påhlman S. (1978). Protamine-agarose non-charged alkyl derivatives of agarose in the purification of rat-liver phosphoprotein phosphatases.. Biochim Biophys Acta.

